# Phases, Microstructures and Mechanical Properties of CoCrNiCuZn High-Entropy Alloy Prepared by Mechanical Alloying and Spark Plasma Sintering

**DOI:** 10.3390/e21020122

**Published:** 2019-01-29

**Authors:** Yuchen Sun, Boren Ke, Yulin Li, Kai Yang, Mingqi Yang, Wei Ji, Zhengyi Fu

**Affiliations:** State Key Laboratory of Advanced Technology for Materials Synthesis and Processing, Wuhan University of Technology, Wuhan 430070, China

**Keywords:** high-entropy alloy, spark plasma sintering, mechanical alloying, mechanical property, microstructure

## Abstract

In the study, an equiatomic CoCrNiCuZn high-entropy alloy (HEA) was prepared by mechanical alloying (MA) and the phases, microstructures, and thermal properties of the alloy powder were explored. The results suggest that a solid solution with body-centered cubic (BCC) phase and a crystalline size of 10 nm formed after 60 h of milling. Subsequently, the alloy powder was consolidated by spark plasma sintering (SPS) at different temperatures (600 °C, 700 °C, 800 °C, and 900 °C). Two kinds of face-centered cubic (FCC) phases co-existed in the as-sintered samples. Besides, Vickers hardness and compressive strength of the consolidated alloy sintered at 900 °C were respectively 615 HV and 2121 MPa, indicating excellent mechanical properties.

## 1. Introduction

Conventional alloy is generally composed of one or two main elements and a small amount of other elements, to enhance its mechanical properties, such as steel and NiAl intermetallics [1,2]. The emergence of high-entropy alloys (HEAs) [3] has broken this traditional notion. A HEA is loosely defined as alloy composed of more than five principal elements with an equimolar ratio (5–35 at.%). High-entropy alloy has high entropy effect, lattice distortion effect, sluggish cooperative diffusion effect, and cocktail effect. It often has simple solid-solutions or amorphous structure [4]. Well-designed HEAs have good mechanical properties including high hardness, high strength, good corrosion, and wear resistance [5].

HEAs can be prepared by various routes, such as vacuum arc-melting and casting [6,7]. However, these routes are not suitable for HEA systems which contain elements with very different melting points. For example, the melting temperature of Cr is 1000 °C above the atmospheric boiling point of Zn, so some systems such as CoCrNiCuZn high-entropy alloy cannot be synthesized by arc-melting route. Besides, arc-melting is not suitable for industrial manufacturing and final products have some limitations in shape and size [8]. Mechanical alloying (MA) is a convenient route to synthesize nanocrystalline HEAs materials. MA can reduce the preparation cost of nanocrystalline materials [9,10]. In addition, HEAs can be easily consolidated from the as-milled powders with spark plasma sintering (SPS) technique [11,12,13].

In this study, we synthesized the CoCrNiCuZn high-entropy alloy by MA and SPS. The phases, microstructures and mechanical properties of the consolidated alloys were also explored.

## 2. Experimental

Metal powders (Co, Cr, Ni, Cu, and Zn with a purity of more than 99.5 wt.% and a particle size of ~45 μm) were mixed according to the equiatomic composition and milled in a planetary ball-miller (300 rpm for 60 h, argon atmosphere) with stainless steel vials and balls as milling media (a ball-to-powder mass ratio of 20:1). N-heptane was used as the processing controlling agent (PCA) to avoid cold welding and oxidation. The MA process was monitored with an interval of 6 h. After 60 h of ball milling, the powder was consolidated by SPS (Dr. Sinter-3.20 MKII, Sumitomo, Osaka, Japan) at different temperatures (600 °C, 700 °C, 800 °C, and 900 °C) under 30 MPa with dwell time of 10 min in argon atmosphere.

The phases of ball milled (QM-BP, Nanjing Nanda Instrument Plant, Nanjing, China) alloys were characterized by X-ray diffractometer (XRD, Rigaku Ultima III, Tokyo, Japan) with a Cu Kα radiation to investigate the crystal structure. The microstructure was analyzed by a scanning electron microscope (SEM, Hitachi 3400, Tokyo, Japan) and a transmission electron microscope (TEM, JEOL JEM-2010HT, Tokyo, Japan). The thermal analysis of as-milled powder was conducted by a differential scanning calorimeter (DSC, NETZSCH 449C, Selb, Germany) heating the alloy to 1500 °C (5 °C/min) in flowing argon atmosphere. According to the Archimedes principle, the density of HEA was determined. The hardness of sectioned and polished specimens was determined by vickers hardness tester (Wolpert-430SV, Aachen, Germany). The compressive properties at room temperature were determined by a MTS810 testing machine (MTS 810, MTS Systems Corporation, Eden Prairie, MN, USA) with a loading rate of 1 mm/min. The dimensions of sample is 2 mm × 2 mm × 5 mm. The fracture surface was analyzed by SEM. A thin foil of sintered material obtained by mechanical thinning and ion milling was analyzed by TEM. At least 5 measurements were performed to calculate the means of vickers hardness and compressive strength.

## 3. Results and Discussion

### 3.1. Mechanical Alloying of CoCrNiCuZn HEAs

#### 3.1.1. X-Ray Analysis

**Figure 1:**
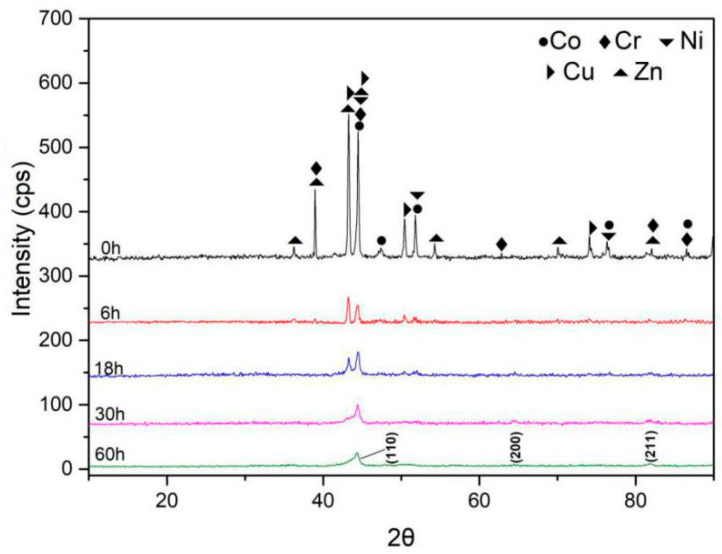
The change of XRD patterns of CoCrNiCuZn high-entropy alloy (HEA) powder obtained after milling for different time (from 0 h to 60 h).

The XRD patterns of the CoCrNiCuZn high-entropy alloy (Figure 1) indicated that a major peak formed after 30-h milling. The diffraction patterns of all alloying elements can be observed in the XRD patterns of primitive blending powder. After 6-h MA, the diffraction peaks of the principle elements were still observed, but the intensity was dramatically decreased. With the increase in milling time to 18 h, some peaks were significantly broadened and some peaks were invisible. After 30-h milling, only 3 peaks of a BCC structure ((1 1 0), (2 0 0), (2 1 1)) could be identified, indicating the formation of a simple solid solution. The BCC solid-solution had a lattice parameter of 2.8831 Å. After 60 h MA, the XRD patterns showed no obvious change. In the milling process, the decreased intensity, broadened or disappeared peak might be caused by high lattice strain, refined crystallite size, and decreased crystallinity [14,15].

The crystallite size (CS) and lattice strain (LS) of CoCrNiCuZn HEA obtained after milling for different time were calculated by Scherrer’s formula after eliminating the interferences of instruments and strain [16,17]. The CS of the BCC phase was significantly refined to 19 nm after 18-h MA and then decreased to 13 nm after 30-h milling (Table 1). Further increasing of milling time had no significant influence on the crystallite size. The equilibrium between crystalline refinement and cold welding of BCC phase might be reached after 30-h milling. The lattice strain of milled powders increased with milling time and reached 0.70% after 60 h milling [18].

#### 3.1.2. Microstructure and Composition

**Figure 2:**
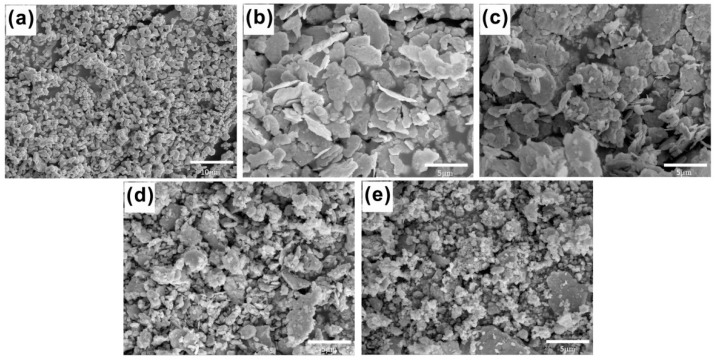
SEM images of CoCrNiCuZn HEA powder obtained after milling for different time: (**a**) 0 h, (**b**) 6 h, (**c**) 18 h, (**d**) 30 h, and (**e**) 60 h.

**Figure 3:**
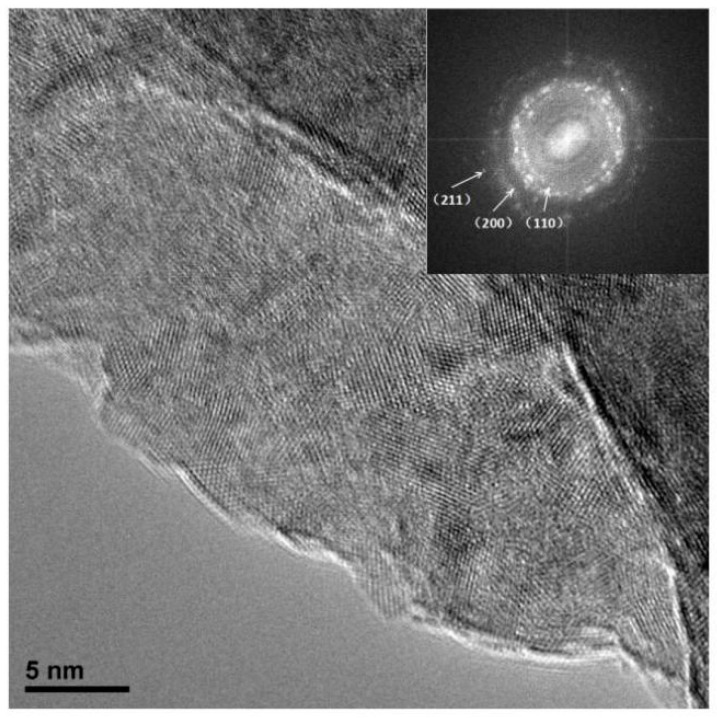
TEM image and selected area electron diffraction (SAED) pattern of CoCrNiCuZn HEA powder obtained after 60 h milling.

Figure 2 shows the microstructure of CoCrNiCuZn HEA powder obtained after ball milling for different time (0 h, 6 h, 18 h, 30 h, and 60 h). The non-milled powder has a different particle size. Through the MA process, milled HEA powder agglomerated into an elliptical shape with the size of ~3 µm and the elliptical particles evolved into ~1 µm thick sheets. The nanocrystalline nature of CoCrNiCuZn HEA obtained after 60 h MA was characterized by the selected area electron diffraction (SAED) pattern and TEM bright field image (Figure 3). The crystal size measured from bright field TEM image was approximately 10 nm, which was consistent with the calculation results by the Scherrer’s formula. The existence of nanoscaled crystallite indicated that the microsized alloy particles in SEM images were the aggregates of nanosized grains.

The rings in the SAED pattern (Figure 3) indicated that the nanocrystalline HEA powder after 60 h milling only consisted of a BCC phase. The result was consistent with XRD analysis results. The results confirmed that the CoCrNiCuZn high-entropy alloy with a structure of simple BCC solid solution had been successfully fabricated by mechanical alloying.

Zhang and Guo proposed the criteria for the formation of solid solution and phase stability of HEA prepared by casting [19,20,21,22,23]. According to the results, the as-calculated values of *ΔS_mix_* (J·K^−1^ mol^−1^), *ΔH_mix_* (kJ·mol^−1^), and *δ* for CoCrNiCuZn HEA were respectively 1.61*R*, 0.96 and 4.4%, which were consistent with the formation criteria of HEAs. Table 2 shows mixing enthalpies of atomic pairs in the CoCrNiCuZn alloy system [24,25]. The main advantage of MA is the extension of solid solubility. Therefore, the simple solid solution is more likely formed in the as-milled HEA than that in the as-cast HEA. The calculated values of *ΔS_mix_*, *ΔH_mix_* and *δ* for CoCrNiCuZn HEA indicated that the simple solid solution should be formed in the MA process.

#### 3.1.3. Thermal Analysis

**Figure 4:**
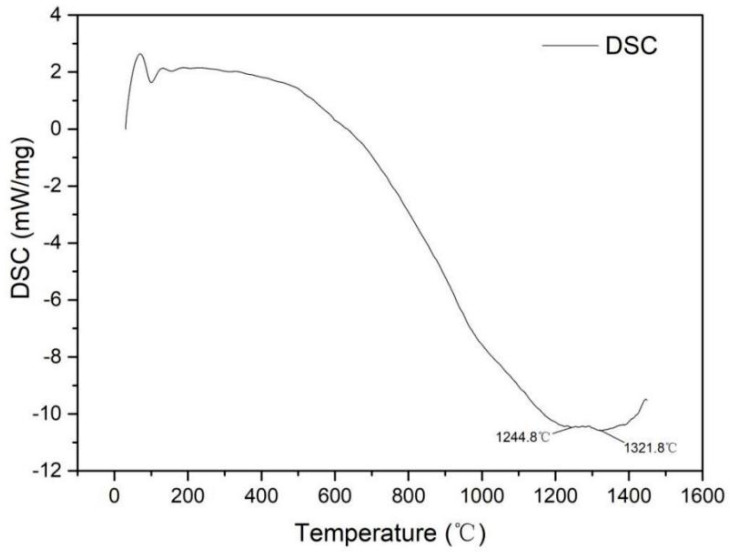
The trend and peaks of the thermal analysis curves (DSC, Mass) of CoCrNiCuZn HEA powder after 60 h ball milling.

Figure 4 shows the DSC results of the CoCrNiCuZn high-entropy alloy powder obtained after 60 h milling. The first endothermic peak at around 100 °C is related to the energy absorption of the PCA evaporation [15]. Then the evaporated matter was eliminated by the flowing argon during testing. In the temperature range of 200~400 °C, the curve was relatively stable. When the temperature was above 600 °C, an endothermic line is observed, indicating that phase changes started at around this temperature. Two endothermic peaks at 1244.8 °C and 1321.8 °C were considered as the melting points of different phases [26], proving that there were two phases after the phase change occurred.

### 3.2. Consolidation by SPS

#### 3.2.1. X-Ray Analysis

**Figure 5:**
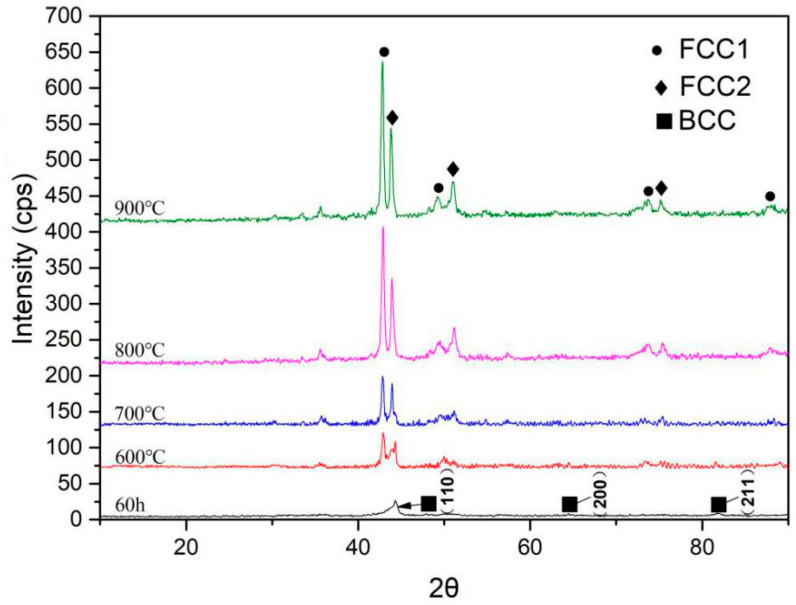
XRD patterns of CoCrNiCuZn HEA powder after 60 h ball milling and CoCrNiCuZn HEA samples fabricated by SPS at different sintering temperatures (600–1000 °C).

Figure 5 shows the XRD patterns of the HEA powder after 60 h ball milling and the samples sintered at 600 °C, 700 °C, 800 °C, and 900 °C, respectively. Two FCC phases were formed at 900 °C and respectively recorded as FCC1 and FCC2. This is consistent with thermal analysis results. 

The above results indicated that both the as-milled CoCrNiCuZn powders and the as-sintered CoCrNiCuZn samples mainly had simple solid solution structures. This phenomenon can be explained by the Gibbs free energy of mixing defined as:

*G_mix_* = *H_mix_* − *TS_mix_*, (1)

where *H_mix_* is the mixing entropy; *G_mix_* is the Gibbs free energy of the mixture; *S_mix_* is the mixing entropy and *T* is absolute temperature. The entropies of solid solution phases were much higher than those of the intermetallics. The increase in the mixing entropy largely decreased Gibbs free energy. Therefore, especially at high temperatures, the solid solution phases were preferentially formed rather than intermetallics and other complex phases [27].

#### 3.2.2. Microstructure

**Figure 6:**
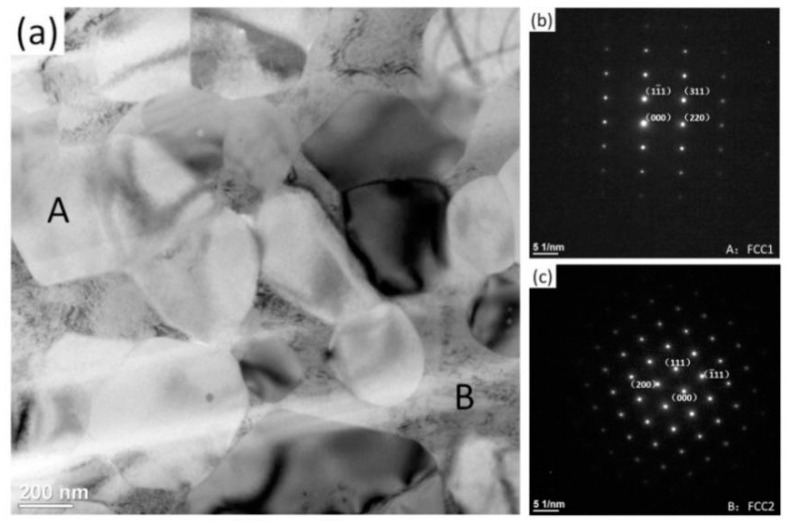
TEM image and SAED patterns of the CoCrNiCuZn HEA bulk obtained after SPS at 900 °C: (**a**) TEM bright field image of bulk CoCrNiCuZn HEA after SPS, (**b**) and (**c**) corresponding SAED patterns respectively indicate Region A with a FCC1 phase and Region B with an FCC2 phase.

The densities of alloys sintered at 600 °C, 700 °C, 800 °C, and 900 °C are respectively 5.26 g/cm^3^, 6.26 g/cm^3^, 7.84 g/cm^3^, and 7.89 g/cm^3^ measured by Archimedes principle. Figure 6 shows TEM bright field image and corresponding SAED patterns of CoCrNiCuZn HEA obtained after SPS at 900 °C. In the TEM image, two different morphologies were observed. Corresponding SAED patterns in Figure 6b,c indicated that the larger particles had a FCC1 structure, whereas the smaller ones had an FCC2 structure. The result was consistent with the XRD results.

**Figure 7:**
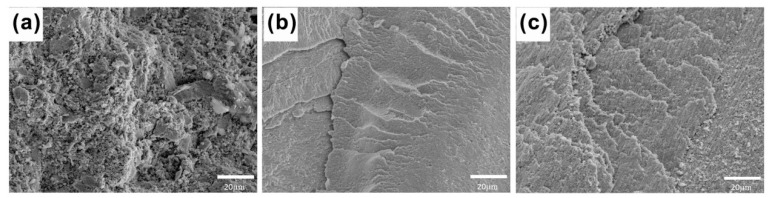
Slip fracture morphology of CoCrNiCuZn HEA samples fabricated by SPS at different sintering temperatures: (**a**) 700 °C, (**b**) 800 °C, and (**c**) 900 °C.

Figure 7 shows the corresponding fractographic feature of the alloys sintered at 700 °C, 800 °C, and 900 °C, respectively. Section structure and stepped structure can be respectively observed in Figure 7a,b. The bulk alloys sintered at 900 °C showed a significant plasticity trend because the FCC phase exhibited a higher plasticity than BCC phase [15].

#### 3.2.3. Mechanical Properties

**Figure 8:**
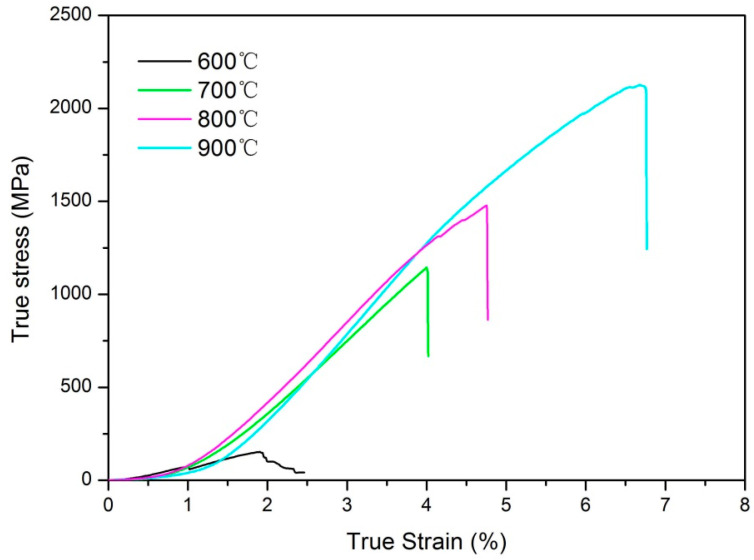
Compressive strain–stress curves at room temperature of CoCrNiCuZn HEA samples fabricated by SPS at different sintering temperatures (600 °C–900 °C).

Figure 8 shows the room-temperature compressive properties of the CoCrNiCuZn HEA consolidated at different temperatures. The strength increases with increasing of sintering temperature. The compressive strength of the sample sintered at 900 °C reached 2121 MPa, which was higher than that of most previously reported HEAs [6,27]. The Vickers hardness of HEA bulk sintered at 900 °C reached 615 HV, which was also superior to most commercial hard facing alloys [28]. The high compressive strength and high hardness are ascribed to the ultrafine grains (as shown in Figure 6a) and solid solution strengthening.

## 4. Conclusions

The equiatomic CoCrNiCuZn HEA powder was successfully synthesized by MA. After 30-h ball milling, a BCC phase structure with a grain size of 10 nm was formed. The thermal analysis curve proved that the BCC phase structure gradually converted into FCC phase at above 600 °C. The XRD and TEM results demonstrated that the high-entropy alloy obtained after sintering had two FCC phases. The sample sintered at 900 °C had a Vickers hardness of 615 HV and a compressive strength of 2121 MPa. The combination of mechanical properties is superior to most of reported HEA systems and commercial hard facing alloys. 

## Figures and Tables

**Table 1:** Crystallite size (CS), lattice strain (LS), lattice parameter (LP) of CoCrNiCuZn HEA obtained after milling for different time (0 h to 60 h).

Milling Time (h)	CS (nm)	LS (%)
0	–	–
6	22	0.64
18	19	0.65
30	13	0.67
60	13	0.70

**Table 2:** Enthalpies (kJ·mol^−1^) between every two elements in CoCrNiCuZn HEA.

Elements	Co	Cr	Ni	Cu	Zn
Co	0	−4	0	6	−5
Cr	-	0	−7	12	5
Ni	-	-	0	4	−9
Cu	-	-	-	0	1
Zn	-	-	-	-	0
